# Current Concepts of Immunology and Diagnosis in Amniotic Fluid Embolism

**DOI:** 10.1155/2012/946576

**Published:** 2011-09-29

**Authors:** Michael D. Benson

**Affiliations:** Department of Obstetrics and Gynecology, Feinberg School of Medicine, Northwestern University, Chicago, IL, USA

## Abstract

Amniotic fluid embolism (AFE) is one of the leading causes of maternal mortality and morbidity in developed countries. Current thinking about pathophysiology has shifted away from embolism toward a maternal immune response to the fetus. Two immunologic mechanisms have been studied to date. Anaphylaxis appears to be doubtful while the available evidence supports a role for complement activation. With the mechanism remaining to be elucidated, AFE remains a clinical diagnosis. It is diagnosed based on one or more of four key signs/symptoms: cardiovascular collapse, respiratory distress, coagulopathy, and/or coma/seizures. The only laboratory test that reliably supports the diagnosis is the finding of fetal material in the maternal pulmonary circulation at autopsy. Perhaps the most compelling mystery surrounding AFE is not why one in 20,000 parturients are afflicted, but rather how the vast majority of women can tolerate the foreign antigenic presence of their fetus both within their uterus and circulation?

## 1. Introduction

When first described in the first half of the 20th century, amniotic fluid embolism was presumed to be the result of the physical obstruction of the maternal pulmonary circulation by fetal material contained within amniotic fluid [1, 2]. In the initial cases described at autopsy, abundant fetal material was seen in pulmonary vessels. The disease is rare, with an incidence ranging from one in 600 to one in 80,000, perhaps because there is no established laboratory marker diagnostic suitable for both survivors and fatalities alike [3, 4]. AFE is one of the leading causes of maternal mortality in the United States and causes roughly 10% of all maternal deaths in developed countries [5–7]. Perhaps an equal concern is the significant maternal morbidity that results in survivors. In 48 British survivors, four had neurological injury, two had thrombotic events, one had renal failure, and another had septicemia [8]. 94% were admitted to the ICU. In an Australian study, three of thirteen survivors suffered a cerebral infarction [9]. The morbidity and mortality for the fetus is also significant. In the British registry, among the fifteen women who died of AFE, eleven of their babies died also, and one of the survivors had hypoxic ischemic encephalopathy [7]. Among the thirty-one surviving women with known newborn outcomes, nine newborns died or suffered serious injury. Although a rare complication of pregnancy, the high rates of injury for both mother and newborn provide compelling arguments for a better understanding of the mechanism of disease.

While “embolism” is contained with the disease name both in its original Spanish and English descriptions, there are a number of confounding clinical observations about AFE that cast doubt on this mechanism of disease [1, 2]. First, mechanical obstruction of pulmonary blood flow is not reliably seen in AFE [10]. Second, clinical events, common in AFE, coagulopathy, adult respiratory distress syndrome, and neurological symptoms, are not typical in pulmonary embolism. Perhaps as a result, observers began speculating about a possible role for an immune mechanism as early as 1950 [11]. Yet with the disease appearing rarely, suddenly, and unpredictably, obtaining relevant evidence in humans has proven unusually difficult. Unfortunately, animal studies have provided little insight other than to suggest there is little to learn from animal models. Illness has been induced in animals (rabbits and dogs) with the intravascular injection of heterologous (human) amniotic fluid contaminated by meconium [2]. In particular, autologous amniotic fluid introduced into the maternal circulation in monkeys seems to be entirely benign [12, 13].

## 2. Anaphylaxis

The first specific suggestion of anaphylaxis as a mechanism of AFE was made by Attwood in 1956 [14]. Benson and Lindburgh suggested that this hypothesis was testable in humans by testing women acutely ill with AFE for serum tryptase [15]. Tryptase, a serine protease with a half-life of several hours, is released by mast cells along with histamine when they degranulate in response to IgE crosslinking on the cell surface in the presence of antigen. Although the specific function of tryptase in anaphylaxis is unknown, with a half life measured in hours instead of the minutes of histamine, the protein has proven useful in the diagnosis of anaphylaxis. Urinary histamine has also been used to diagnose anaphylaxis as a small percentage of histamine is excreted into the urine, unmetabolized [16].

In considering the role of mast cell degranulation in the mechanism of amniotic fluid embolism, the serological evidence should be considered separately from the histological data since, in both cases, the evidence is somewhat mixed. Serum tryptase testing in AFE points against a role for AFE while tryptase staining in pulmonary histology at autopsy provides a more nuanced picture.

In 2001, a study of nine women with amniotic fluid embolism was published in which specific evidence of anaphylaxis was sought [17]. Seven of the nine patients had sera stored as part of the Japanese National Maternal Mortality Surveillance project while two patients were American survivors enrolled in a protocol specifically created to collect acute samples in AFE patients. Five patients were negative for serum tryptase, one was negative for urinary histamine, and one was negative for both. There were no positive findings for either serum tryptase or urinary histamine in any of the patients.

Since this study, elevated serum tryptase levels have been reported in fatal cases of amniotic fluid embolism. In three such reports, serum tryptase levels have been only slightly elevated, raising serious questions about the premise that anaphylaxis reflects the underlying mechanism [18–20]. In the fourth case of AFE, the authors also looked at postmortem serum tryptase levels in nonpregnant, non-AFE cases such as pulmonary embolism and traffic accidents [21]. While the serum tryptase level in the AFE case was six times the upper level of normal, 17 hours after death, the one confirmed anaphylaxis case had a tryptase levels over 60 times normal, 24 hours after death. Finally tryptase levels in a fifth, surviving case of AFE were normal [22]. The slight serum elevations in fatal cases of AFE are below that normally seen for postmortem values seen in anaphylaxis and do not support the hypothesis that mast cell degranulation plays a central role in the pathophysiology of amniotic fluid embolism.

With 100% of 6 AFE patients testing negative for tryptase in the largest series to date, the best that can be said about serum tryptase in AFE is that it has very poor sensitivity [17]. However, there are objections to the specificity of the test as well. Unless tryptase is measured in both normal parturients and mortally ill obstetric patients with illnesses other than AFE, the specificity of tryptase will remain unknown. It is possible that tryptase is elevated in specific obstetric populations without AFE. At the present, serum tryptase in AFE can only be considered an investigational tool with both normal and elevated levels neither confirming nor excluding the diagnosis.

The evidence from maternal pulmonary histology is more complex concerning the role of anaphylaxis in AFE. Two studies by Italian pathologists did find evidence of mast cell degranulation in the maternal pulmonary vasculature. The first study performed mast cell counts per fixed area by using an immunohistochemical stain for tryptase [23]. The investigators compared the six fatal AFE cases with six anaphylactic deaths, five traumatic pregnancies deaths, and six traumatic deaths in nonpregnant women. Remarkably, the AFE group had higher mast cell densities than the anaphylactic group and much higher than the other two control groups. In the second study using a similar approach, the investigators were able to show a large increase in extracellular tryptase consistent with mast cell degranulation in the eight women in the AFE group compared to the six pregnant women who succumbed from traumatic injury [20]. One case report of AFE failed to find immunohistochemical evidence of mast cell degranulation at autopsy, but the patient in question died minutes after a head on motor vehicle accident and did not clearly have AFE [24].

In summary, the available evidence suggests that mast cell degranulation does take place in the lungs in fatal AFE cases and does not do so in other mortal pregnancy conditions. This is hard to reconcile with the general lack of significant circulating tryptase elevations in AFE. These observations together suggest that pulmonary mast cell involvement in fatal cases may be a secondary process and not necessarily the primary mechanism of AFE. This possibility is all the more intriguing because mast cell degranulation can occur as a result of complement activation [25]. In considering the mechanism of disease, evidence of tryptase release cannot be taken as proof as a primary role for anaphylaxis without the simultaneous measurement of complement and confirming that activation did not take place.

Finally before leaving the subject of anaphylaxis and amniotic fluid embolism, the suggestion that the disease be renamed “Anaphylactoid Syndrome of Pregnancy” should be considered [26, 27]. While the term “anaphylaxis” can refer loosely to the clinical symptoms resulting from any process resulting in mast cell degranulation, it is more commonly used to denote a process mediated by IgE binding to antigen [28]. In contrast, the common usage of “anaphylactoid” is used specifically to refer to nonimmune-mediated degranulation of mast cells—not involving IgE [29]. The best known example in humans of an “anaphylactoid” reaction is the histamine release that is sometimes seen in people with their first exposure to intravenous X-ray contrast [30]. Potentially as fatal as IgE-mediated anaphylaxis, the presumption underlying anaphylactoid reactions is that they do not result from a remembered antigenic response since there has not been previous exposure. Beyond the fact that mast cell degranulation is by no means a proven mechanism of AFE is the idea that all speculation regarding disease mechanism centers on some type of maternal immune response to fetal antigen [31]. No mechanism has been proposed for AFE in which mast cells degranulate in the absence of antibody-antigen interaction. Although “Anaphylactoid Syndrome of Pregnancy” has not gained widespread acceptance, available evidence does not support changing the name of AFE, and the term should be abandoned.

## 3. Complement Activation in AFE

The frequency of adult respiratory distress syndrome as a sequela of AFE suggested to Jacob and Hammershmidt in 1982 [32], and Hammershmidt et al. [33] that complement activation may have a role to play in the pathophysiology of disease. The first series in which serum complement levels were evaluated in AFE patients found that both C3 and C4 were significantly depressed in all eight AFE patients in which measurements could be done [17]. A control group of twenty-three normal laboring patients all had complement levels in the normal range. Furthermore, in a case report concerning possible “mild” AFE in which a patient had transient shortness of breath, palpitations, and laboratory evidence for a coagulopathy, complement levels were also depressed [34]. However, in a separate series of AFE patients in Japan, the average C3 level was low normal (71 mg/dL) while the average C4 levels were depressed (13.9 mg/dL) [7]. There were no differences in the means between those who survived and those who died. The Italian group that did histology studies for tryptase also considered complement activation in their most recent paper [20]. They found depressed amounts of C3a in the pulmonary circulation of the eight AFE patients compared to the six pregnant women dying of trauma. The investigators suggested that the diminished pulmonary C3a was consistent with complement activation. As noted previously, there was evidence for tryptase release in the same histology samples. It is possible, if not likely, that complement activation may have been the initial immune response which then resulted in a secondary degranulation of mast cells. 

With both serologic and histologic evidence, the complement pathway appears more promising than anaphylaxis as a possible mechanism of disease. However, as a diagnostic tool, both sensitivity and specificity are poorly characterized. As with tryptase, complement levels in AFE remain an investigational tool and should not be used to either confirm or refute a clinical diagnosis.

During the investigations into complement activation in AFE, an interesting footnote should be mentioned. It appears that some degree of complement activation during normal labor is physiologic and peaks at the moment of birth. Two separate studies in which serial complement levels were obtained in normal laboring parturients have been performed. The first was done as part of the 2001 study looking at complement and tryptase levels in AFE [17]. A healthy control group of thirteen American women and nine Japanese women had complement levels obtained on admission to the hospital in labor and on the first postpartum day. A within pairs analysis was performed, and both C3 and C4 demonstrated statistically significant declines, 8% and 5%, respectively. In a second study of twelve healthy American women, serial complement levels were obtained several times during labor and within an hour postpartum [35]. Again, the decreases in complement levels were highly statistically significant with C3 dropping an average of 15% and C4 diminishing by 11%. In both studies, all complement levels remained within the normal range. Taken together, they suggest that complement activation peaks at or shortly before birth and that levels start to return to normal in the postpartum period [10]. The biological significance of complement activation during normal parturition is unknown but is consistent with current evidence that the initiation of labor may be mediated by an inflammatory response rather than simply a fluctuation in hormones [36].

## 4. Clinical Diagnosis of AFE

There is a reasonably broad consensus in the published literature on the clinical diagnosis of AFE without the reliance on laboratory markers. In general, the definitions of the disease point to one or more of the following clinical signs first characterized by Courtney in 1974: cardiovascular instability, respiratory distress, coagulopathy, or coma/seizures in the absence of other explanations [37]. Benson used the same definition for his research protocols but stipulated a forty-eight hour time limit from birth [10]. The Japan Consensus Criteria for the diagnosis of AFE was similar with the omission of neurological symptoms and a twelve-hour limit from birth [7]. Conde-Agudelo and Romero of the National Institutes of Health adopted the Benson definition for clinical diagnosis in their 2009 review article, which was also used in an Australian population-based cohort study [9, 38]. In sum, the clinical definition of AFE has remained reasonably constant over the past four decades.

The UK Obstetric Surveillance System criteria for AFE are a bit more nuanced although still clearly similar to the Japanese and the American clinical definitions [39]. The British do not define a time limit from parturition and include maternal hemorrhage, in general, as well as coagulopathy. Unlike the other clinical definitions, they do consider acute fetal compromise in the absence of other clear cause as a diagnostic sign of AFE. They also include nonspecific premonitory symptoms such as restlessness or numbness, agitation, and tingling. Perhaps most significantly, the British include one laboratory test as being sufficient for the diagnosis regardless of clinical course—the finding of fetal material in the maternal pulmonary vasculature at autopsy.

There are several nuances to the clinical diagnosis of AFE. All definitions assign the diagnosis with the proviso that alternative diagnoses have been excluded although this does not preclude other comorbidities such as abruption. Yet several other clinical findings are associated with AFE even if they are not always considered diagnostic in their own right. For instance, uterine atony has been closely associated with AFE in several reports [37, 40]. Beyond being a diagnostic criteria in the United Kingdom, several case reports have also described fetal bradycardia occurring early in the onset of amniotic fluid embolism [6, 41, 42]. Regarding coagulopathy and the diagnosis of amniotic fluid embolism, at least six case reports have described coagulopathy alone as the sole clinical sign/symptom of AFE [43–48]. It should also be noted that all clinical definitions of the disease described above anticipate that coagulopathy occurring in isolation may be the sole clinical sign/symptom of AFE [7, 10, 37–39].

## 5. Laboratory Diagnosis of AFE

An examination of the current state of laboratory diagnosis of AFE is fraught with conflicting and paradoxical evidence. To be sure, AFE as a clinical entity emerged from the autopsy room with eight maternal deaths at the University of Chicago defining the first series of the disease. These first cases had large amounts of apparent fetal material in the maternal pulmonary vasculature. It is, therefore, no surprise that finding any fetal material in the maternal circulation has been presumed to confirm a diagnosis of AFE. However, this is clearly not the case as there have been any number of both individual case reports and case series in which fetal material is present in the maternal circulation in women not experiencing AFE. To add confusion to the issue, the limited autopsy studies available draw quite a different picture from studies in living patients.

There are a growing number of reports in the literature in which fetal material is found in the maternal circulation of living parturients who do not have amniotic fluid embolism. Perhaps the earliest reports are those of two separate cases in which fetal material was found in the maternal circulation of the uterus at the time of peripartum hysterectomy in women who did not have AFE [49, 50]. In the 1980s, at least three studies were done in which pulmonary artery samples were taken from critically ill pregnant patients, looking for fetal material [51–53]. Any number of these aspirates had fetal material, ranging from squamous to mucin to lanugo in women who did not have AFE. This is further supported by a recent pathology study of 57 peripartum hysterectomy specimens for women who were experiencing excessive bleeding [54]. There was no relationship between the presence or absence of uterine intravascular fetal material and the etiology of maternal hemorrhage. Seven diagnoses were attributed to the etiology of the hemorrhage: uterine rupture, abruption, uterine atony, placenta previa, accreta, coagulopathy, and retained placenta. Specifically, women in all seven diagnostic categories had both the presence and the absence of fetal material including those with disseminated intravascular coagulopathy, one of the signs of amniotic fluid embolism.

In contrast, two case reports relied on the observation of intravascular fetal material in the uterus to confirm a diagnosis of amniotic fluid embolism. In the first, the authors assume that they “prevented AFE” by ligating the uterine vessels with the fetal material in it [55]. They saw vernix and air bubbles entering the uterine vein and ligated the uterine vessels without removing the uterus. The patient's only morbidity was a subclinical laboratory coagulopathy. In the second case report, the authors similarly assume that a fatal case was averted because the fetal material did not enter the pulmonary circulation [43]. They observed fetal material within the uterine veins after a peripartum hysterectomy on histology studies. In light of the numerous reports cited previously, the currently available evidence does not support using the presence or absence of intravascular fetal materials in living patients to confirm or refute a diagnosis of AFE.

To make matters more confusing, the limited data from autopsy studies suggest that intravascular fetal material is both sensitive and specific for AFE. In 1998, Japanese investigators used a immunohistochemical stain for a fetal antigen, STN, to make a diagnosis of AFE at autopsy [56]. All four AFE patients had positive staining of the pulmonary vasculature while the control group of four women who died from non-AFE causes did not. A second study of twenty-seven maternal deaths found fetal material in 100% of AFE patients and none in those dying of non-AFE causes [57]. It should be noted that the study findings were problematic since the authors began with the assumption that any fetal material in the maternal vasculature was diagnostic of AFE. With that said, they did not find fetal material in any cases that were obviously not AFE such as traumatic injury. The previously cited Italian studies also found fetal material in the pulmonary vasculature in AFE patients at autopsy while not finding it in pregnant women dying of other causes [20, 23]. The evidence from several dozen autopsies on women with and without AFE suggests that the finding of fetal material in the maternal circulation at autopsy is specific for AFE. This fact is utilized in the diagnostic criteria of the United Kingdom as the only laboratory test considered diagnostic [39]. Similarly, histology at autopsy seems to be sensitive for AFE although, in all studies, specialized stains were used to look for fetal material.

The difference in the presence of fetal material in the maternal vasculature between those dying and surviving is seen in another study of 135 women with AFE in Japan [7]. In this study, serum levels of a specific fetal antigen, STN, were higher in those succumbing to the disease. However, the levels between survivors and those dying did overlap sufficiently to prevent STN levels alone from being a very useful, discriminating diagnostic marker for AFE.

As with tryptase and complement, the presence or the absence of fetal material in the maternal circulation of living women cannot either confirm or refute the diagnosis of AFE. However, the limited available evidence suggests a somewhat different conclusion at autopsy, where the presence of intravascular fetal material does seem to be specific for AFE. It is unclear why there should be a difference in the sensitivity and specificity of intravascular fetal material between the living and the dead. An obvious explanation is that a greater amount of fetal material may be more mortal and thus more detectable at autopsy. Yet this does not seem entirely satisfactory on further examination since the amount of fetal material in fatal AFE cases was not necessarily massive and indeed present only in microscopic amounts.

## 6. Conclusions

Much has been published about amniotic fluid embolism that, in light of current evidence, needs to be revised. The evidence does not support renaming the disease “Anaphylactoid Syndrome of Pregnancy.” The only laboratory test that is diagnostic at this time is the finding of fetal material in the maternal pulmonary circulation at autopsy. Serum tryptase levels should not be used to affirm or refute a diagnosis of AFE. Similarly, while there is better evidence concerning complement, it too remains an investigational tool and is not yet a reliable diagnostic marker. There is enough of a worldwide consensus on diagnostic criteria to permit reasonably consistent clinical diagnoses in the absence of confirmatory laboratory testing at the present. Generally, the diagnosis relies on one or more of four signs or symptoms occurring during pregnancy or shortly thereafter: cardiovascular collapse, respiratory distress, coagulopathy, and coma or seizures. Finally, intravascular fetal material in living women clearly does not necessarily result in AFE although it may not be either benign or physiologic. Perhaps the best way forward is the continued use of a difficult but achievable methodology—looking at immune markers in sera obtained from healthy controls and acutely ill pregnant women, both with and without AFE. The study of AFE is important both because it causes significant morbidity and mortality among mothers and their babies and also because a better understanding of this disease may lead to a more insight into immune tolerance in general. The overarching importance of the question underlying the mechanism of AFE is not so much why does a specific patient get the disease but rather how can women tolerate the presence of so much foreign antigen within both their uterus and their circulation?
